# Enhanced co-culture and enrichment of human natural killer cells for the selective clearance of senescent cells

**DOI:** 10.18632/aging.203931

**Published:** 2022-03-04

**Authors:** Kristie Kim, Tesfahun Dessale Admasu, Alexandra Stolzing, Amit Sharma

**Affiliations:** 1 SENS Research Foundation, Mountain View, CA 94041, USA; 2 Loughborough University, Centre for Biological Engineering, Wolfson School of Electrical, Material and Manufacturing Engineering, Loughborough, UK

**Keywords:** aging, senescence, natural killer cells, NKCC, immune surveillance

## Abstract

In the context of aging and age-associated diseases, Natural Killer (NK) cells have been revealed as a key cell type responsible for the immune clearance of senescent cells. Subsequently, NK cell-based therapies have emerged as promising alternatives to drug-based therapeutic interventions for the prevention and treatment of age-related disease and debility. Given the promise of NK cell-mediated immunotherapies as a safe and effective treatment strategy, we outline an improved method by which primary NK cells can be efficiently enriched from human peripheral blood across multiple donors (ages 20-42 years old), with a practical protocol that reliably enhances both CD56^dim^ and CD56^bright^ NK cells by 15-fold and 3-fold, respectively. Importantly, we show that our co-culture protocol can be used as an easily adaptable tool to assess highly efficient and selective killing of senescent cells by primary NK cells enriched via our method using longer co-culture durations and a low target to effector ratio, which may be more physiological than has been achieved in previous literature.

## INTRODUCTION

Advanced age is the greatest risk factor for most chronic diseases, with over 90% of adults aged 65 or older experiencing at least one chronic disease such as cancer, diabetes and cardiovascular disease [1]. Progressively increasing numbers of these adults are suffering from multimorbid age-related conditions [2]. Many aging phenotypes and pathologies, including diverse age-associated diseases and disorders, are causally linked to the accumulation of senescent cell burden with increasing age [3, 4]. Senescent cells are characterized by an irreversible cell-cycle arrest of proliferation-competent cells, along with morphological and metabolic changes, altered gene expression, chromatin reorganization, and a complex pro-inflammatory senescence-associated secretory phenotype (SASP), which contributes to chronic inflammation and damage to surrounding cells and tissues [4, 5]. Senescent cells are thought to be cleared by the immune system, but increasing age or disease severity allows senescent cells to escape the process of immunosurveillance and accumulate in older individuals [6]. The removal of senescent cells via drugs that selectively kill senescent cells (“senolytic” drugs) or genetic manipulation in transgenic mouse models can prevent or delay tissue dysfunction, improve age-related pathologies, and extend health span, suggesting that a reduced senescent cell burden in aging adults merits further study as a therapeutic target for the treatment and prevention of disease of aging [7, 8].

However, exploiting the ability of the innate immune system to surveille senescent cells has emerged as an alternative approach for their elimination [9]. Several lines of evidence show that Natural Killer (NK) cells play a vital role in the targeted elimination of senescent cells [9–11]. In fact, regulation of senescence burden by NK cells is not only considered essential for tissue homeostasis [12, 13], but also has been shown to be important for the regulation of pathological states such as tumor growth [11, 14]. Moreover, impairment of NK cell function has been shown to accelerate aging in perforin-deficient mice due to the accumulation of senescent cells [15].

Although senescent cells are known to secrete chemokines as a part of the SASP to attract NK cells, they have also evolved strategies to evade clearance by NK cells [16]. For instance, senescent fibroblasts can escape immune surveillance by increasing expression of HLA-E, which upon interaction with NKG2A inhibits NK cell cytotoxicity [17]. Furthermore, senescent cells can shed MICA and MICB (ligands for the NK cell-activating receptor NKG2D), preventing the binding of NK cells to their targets [18, 19]. Thus, whether through NK cell-based adoptive cell therapies or removal of NK cell inhibitory ligands, there is tremendous promise in modulating NK cells as an intervention against age-related diseases [9].

However, an impediment to the development of NK cell-based senotherapeutic strategies is that although several publications have demonstrated the immune surveillance potential of NK cells towards senescent cells, the co-culture strategies employed do not necessarily reflect physiological conditions. For instance, many studies demonstrating immune surveillance of senescent cells by NK cells used very high target to effector (T:E) ratios. It has been shown that most NK cell killing occurs through serial killing whereby a single NK cell can kill up to 10 targets [20]. Furthermore, a single IL-2-activated NK cell releases about a tenth of its total lytic granule reserve after 16 hours of co-culture [21, 22]. However, human YT cells (human NK cell line) have been used with T:E ratios as high as 1:20 to achieve modest cytotoxicity towards senescent IMR-90 cells [11, 23, 24]. Lannello et al. reportedly used T:E ratios as high as 1:81 [25]. Interestingly, although Pereira et al. developed an autologous co-culture system with skin-derived primary human fibroblasts and NK cells, cytotoxicity towards senescent fibroblasts was low (10-20%) even with a high T:E ratio of 1:20 [17, 24, 25]. In addition, in most of these studies, co-culture experiments were performed for relatively short (2-6 hours) durations [11, 17, 23–25]. Finally, Interleukin-2 (IL-2) is a cytokine widely known to induce proliferation and activation of resting NK cells and is vital for their cytotoxic function both in cell culture and *in vivo* [26]. However, the concentration of IL-2 used to demonstrate the ability of NK cells to kill senescent cells varies substantially [18, 21, 27, 28].

In the present study, we demonstrate an easily adaptable and more physiological co-culture system in which we use freshly isolated peripheral NK cells cultured for 3 days with a relatively low concentration (100 IU/ml) of human recombinant Interleukin-2 (rIL-2). We have shown that even with different target cell types, NK cell donors, and methods of senescence induction, our co-culture method is robust and reliable, consistently achieving two- to three-fold higher cytotoxicity of NK cells towards senescent cells compared to non-senescent cells at a co-culture duration of 16 hours and low 1:1 T:E ratio. Our protocols for NK cell isolation and enrichment and co-culture may more fully capture the interaction between senescent cells and NK cells than has been previously achieved. Importantly, this simple, robust protocol may serve as a platform for the development of novel NK cell-based senescent cell ablation strategies.

## RESULTS

### Genotoxic stress-induced model of senescence

**Figure 1:**
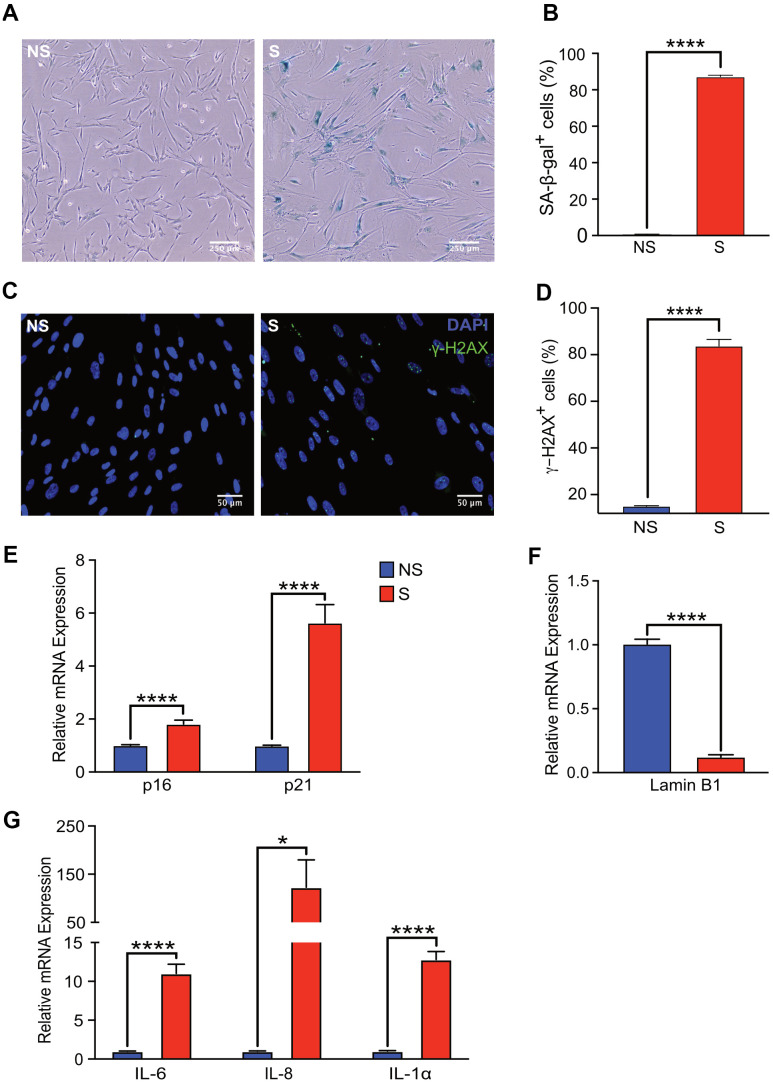
**Senescent human fibroblasts express markers of senescence.** IMR-90 fibroblasts were induced to senesce by doxorubicin (300 nM, 24 h) and SA-β-Gal staining was performed on day 9 after doxorubicin treatment. (**A**) Representative images of SA-β-Gal stained senescent (S) and non-senescent (NS) cells. (**B**) Quantification of SA-β-gal-positive cells in NS and S IMR-90 cells. Four fields were quantified per well (n=6) with a total of 7713 and 2666 cells counted for NS and S cells, respectively. (**C**) Immunofluorescence was performed to detect γ-H2AX in NS and S IMR-90 cells, 10 days after exposure to vehicle or doxorubicin, respectively. Representative images of NS and S cells stained for γ-H2AX (green) and Hoechst (blue). (**D**) The percentage of cells with 3 or more γ-H2AX foci/cell (γ-H2AX+ cells) was scored from a total of 780 NS and 387 S cells. The results are presented as mean % of cells with 3 or more foci/cell. (**E**–**G**) mRNA levels of cell cycle regulators *p16* and *p21*, *Lamin B1*, and various SASP factors, *IL-6*, *IL-8*, and *IL-1α* assessed through Quantitative Realtime PCR in NS and S IMR-90 cells (n=3). All results are presented as a mean and error bars represent ±SEM. Statistical analysis performed using unpaired t test. *p < 0.05, **p < 0.01, and ***p < 0.001.

Human fetal lung fibroblasts (IMR-90) cells were used as a cell culture model of cellular senescence. Robust induction of SA-β-gal activity was observed in 87% of doxorubicin-treated (300 nM) cells, nine days after treatment whereas 1% of non-senescent control cells showed SA-β-gal activity (Figure 1A, 1B). In addition to doxorubicin treatment, additional models of senescence induction such as irradiation (20 Gy) and treatment with the mitochondria damaging agent, etoposide (20 μM, 48 h) were tested. A statistically significant percentage of cells with increased SA-β-gal activity was observed in cells exposed to x-rays or etoposide (87% and 81%, respectively) nine days after treatment compared to non-senescent control cells (1%) (Supplementary Figure 1B, 1C).

The increase in SA-β-gal activity of doxorubicin-treated fibroblasts correlated with persistent DNA damage, as measured by immunofluorescence staining for γ-H2AX (Figure 1C). The percentage of senescent cells with >2 γ-H2AX foci per cell was eight-fold higher compared to that of non-senescent cells (Figure 1D). As expected, mRNA levels of cell cycle checkpoint markers p16^INK4A^ and p21^CIP1^ were significantly elevated in senescent compared to non-senescent cells (Figure 1E). Loss of high mobility group box 1 (HMGB1), a highly conserved nuclear protein from senescent cells, is often used as a biomarker of senescence [29]. The loss of HMGB1 from the nucleus of our senescent fibroblasts was confirmed in doxorubicin-treated cells (Supplementary Figure 1A). Further, loss of Lamin B1 expression, another hallmark of senescence [30], was also observed in senescent compared to non-senescent cells (Figure 1F). Finally, increased mRNA levels of classical SASP factors IL-6, IL-8 and IL-1α in senescent cells was confirmed by qRT-PCR [31, 32] (Figure 1G). Collectively, these findings validated robust induction of a senescence phenotype in our cell culture model.

### Isolation and enrichment of NK cells

**Figure 2:**
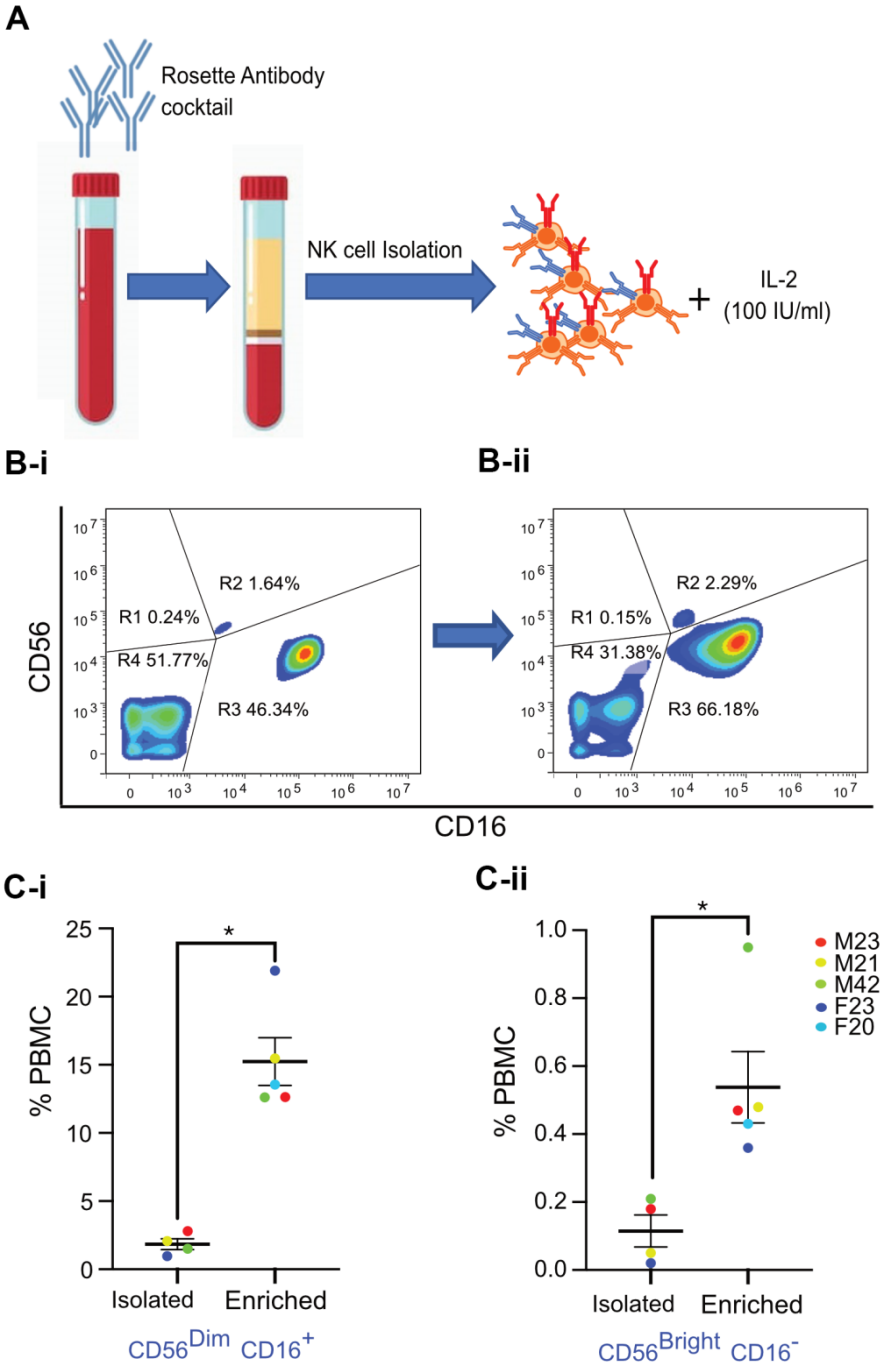
**Isolation and enrichment strategy of primary NK cells from human PBMC.** (**A**) Experimental design of the NK cell enrichment strategy. PBMCs were collected from multiple donors (ages 20-42 years old), and NK cells were isolated and enriched. (**B**) Flow cytometry analysis of CD56 and CD16 expression in NK cells before and after enrichment for a representative donor. (**B-i**) Before enrichment, 1.64% of NK cells (0.18% of PBMCs) were CD56^Bright^ CD16^-^ and 46.34% of NK cells (2.8% of PBMCs) were CD56^Dim^ CD16^+^ NK cells. (**B-ii**) After enrichment, 2.29% of NK cells (0.47% of PBMCs) were CD56^Bright^ CD16^-^ and 66.18% of NK cells (12.6% of PBMCs) were CD56^Dim^ CD16^+^ NK cells. (**C-i**) Average percentage of CD56^Dim^ CD16^+^ NK cell population in PBMCs from five donors. (**C-ii**) Average percentage of CD56^Bright^ CD16^-^ NK cell population in PBMCs from five donors. Donor sex and age are indicated in the figure. Statistical analysis performed using paired t test. *p < 0.05, **p < 0.01, and ***p < 0.001.

Freshly drawn whole blood (40 ml) was treated with RosetteSep human NK cell cocktail (Stem Cell Technologies, USA), which removes unwanted cells with Tetrameric Antibody Complexes that binds to white blood cells (except NK cells) and crosslinks them to red blood cells (RBCs). Following density gradient centrifugation, the NK cell population at the interface between the plasma and buoyant density medium was isolated. Freshly isolated NK cells were then incubated in RPMI media supplemented with 20% FBS and 100 IU/ml rIL-2 for 72 hours for enrichment (Figure 2A).

The relative expression of CD56 and absence of CD3 are routinely used to identify human NK cells [33]. Low (CD56^dim^) and high (CD56^bright^) CD56 expression levels define major subsets of NK cells [33]. CD56^bright^ CD16^-^ cells are considered immature NK cells that secrete interferon-γ (IFNγ), whereas CD56^dim^ CD16^+^ NK cells are responsible for cytotoxicity [34]. Upon physical interaction with target cells, cytotoxic NK cells release perforin, granzymes (serine proteases), and other cytotoxic granules that kill their targets [35]. FACS analysis was performed to characterize NK cells freshly isolated from donors between the ages of 20-42 years. Results revealed a classic distribution of CD56^bright^ CD16^-^ and CD56^dim^ CD16^+^ NK cells, as evidenced by CD16 versus CD56 flow cytometry analysis in the scatter plots of fresh human Peripheral blood mononuclear cells (PBMCs) exemplified by one donor (after gating out CD3+ T cells as shown in Figure 2B-i and Supplementary Figure 2B). Specificity of antibodies was confirmed by Fluorescence minus one (FMO) control experiments with NK cell populations (Supplementary Figure 2C-i, 2C-ii). Interestingly, statistically significant enrichment of both sub-populations of NK cells was observed after culturing cells in media with rIL-2 for three days (Figure 2B-ii). This increase in the numbers of NK cell subtypes was consistent in NK cells isolated and enriched from multiple donors, with a fifteen-fold increase in the numbers of CD56^dim^ CD16^+^ NK cells and approximately three-fold increase in the numbers of CD56^bright^ CD16^-^ NK cells (Figure 2C-i, 2C-ii).

### NK cell-mediated killing of senescent cells

**Figure 3:**
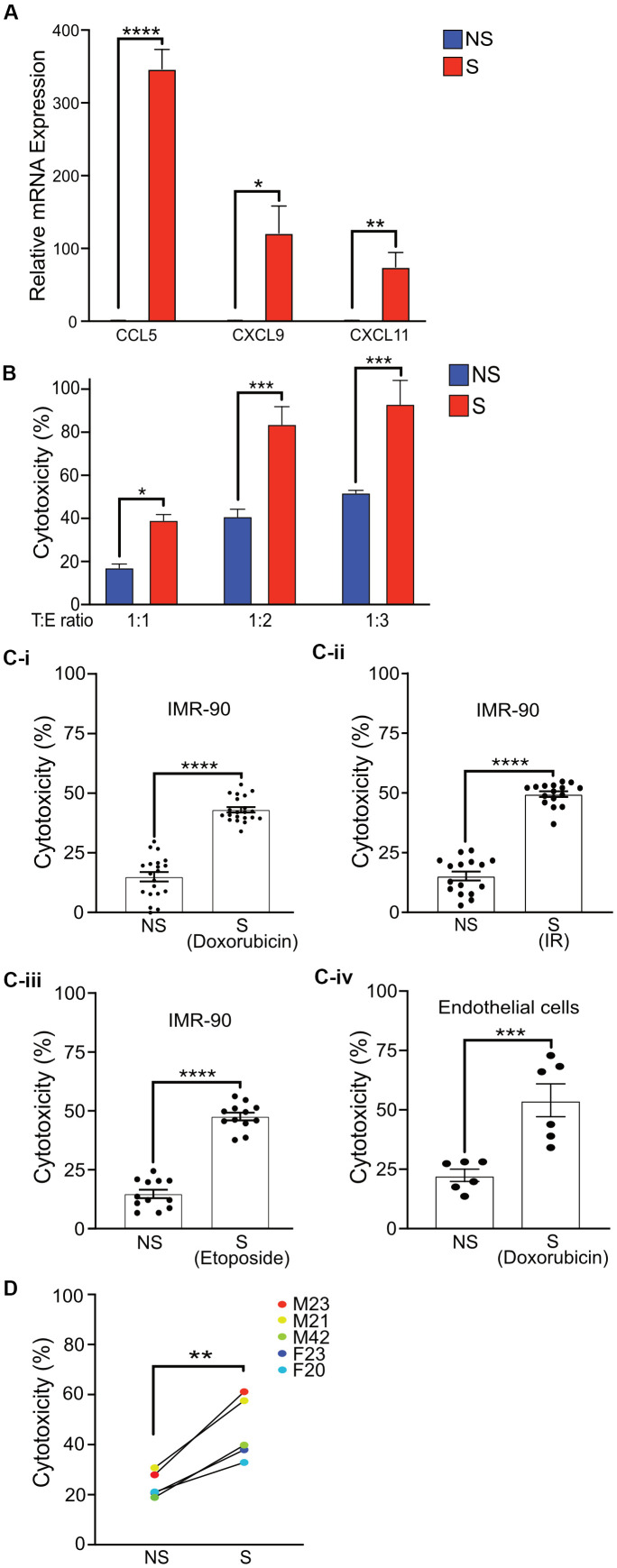
**Activated primary NK cells selectively eliminate senescent cells.** (**A**) Quantitative Realtime PCR was performed to detect the mRNA levels of *CCL5*, *CXCL9*, and *CXCL11* in non-senescent and senescent IMR-90 fibroblasts. The results are presented as mean fold change in NS compared to S samples from two independent experiments performed in triplicate, and error bars represent ±SEM. Statistical analysis performed using unpaired t test. *p < 0.05, **p < 0.01, and ***p < 0.001. (**B**) NS or S IMR-90 fibroblasts were co-incubated with NK cells for 16 h at T:E ratios of 1:1, 1:2 and 1:3 and cytotoxicity was evaluated by LDH release. The graphs show the mean and S.E. of % LDH release. NS or S (**C-i**) doxorubicin-treated (n=6), (**C-ii**) irradiated (n=3) or (**C-iii**) etoposide-treated (n=3) IMR-90 fibroblasts or (**C-iv**) doxorubicin-treated endothelial cells (n=2) were overlayed with NK cells for 16 hours at T:E ratio of 1:1, and cytotoxicity was evaluated by LDH release. The results are plotted as mean % cytotoxicity for NS and S cells with each experiment performed in at least triplicate. The graphs show mean % LDH release. (**D**) NK cells isolated and enriched from three different individuals were co-cultured with NS or S IMR-90 cells at T:E ratio of 1:1 and cytotoxicity was evaluated by LDH release after 16 hours of co-culture. Experiments were performed in triplicate and the results are plotted as mean % cytotoxicity for NS and S. Donor sex and age are indicated in the figure. Statistical analysis performed using unpaired t test. *p < 0.05, **p < 0.01, and ***p < 0.001.

Senescent cells are known to secrete a variety of factors as part of the SASP that can attract NK cells and either activate or inhibit cytotoxic function [9, 14, 36]. To test whether senescent cells in our senescence model produced increased levels of these factors, we measured mRNA expression of multiple cytokines. A robust and statistically significant increase in the mRNA levels of CCL5, CXCL9 and CXCL11, which are known chemoattractants for NK cells, was observed in senescent cells compared to non-senescent controls (Figure 3A). A modest increase in mRNA expression of CCL2, decreased expression of CXCL12, and non-significant difference in Chemerin compared to non-senescent cells was also observed (Supplementary Figure 2A).

Although the role of NK cells in targeting senescent cells has been previously demonstrated, the protocols used are quite variable and employ conditions that may not be as physiologically representative. Hence the cytotoxic potential of NK cells isolated and enriched with our protocol for the selective killing of senescent cells was determined by lactate dehydrogenase (LDH) release. Even at a T:E ratio of only 1:1, NK cells selectively eliminated senescent fibroblasts at a significantly higher (43%) level compared to non-senescent control cells (15%) after 16 hours of co-culture. In addition, twice (83%) as many senescent fibroblasts were killed by NK cells when the T:E ratio was doubled to 1:2 compared to 41% in NS cells, and further increasing the T:E ratio to 1:3 killed nearly all senescent fibroblasts (93%) compared to 51% in NS cells. However, at higher T:E ratios of 1:2 and 1:3, substantial killing of non-senescent cells was also observed (Figure 3B and Supplementary Figure 3A).

Recent publications have reported substantial variability among senescent cells based on mode of senescence induction and cell type [37]. Thus, whether NK cells enriched by our protocol can eliminate senescent cells induced to senesce by different stressors was investigated. NK cells killed 43-50% of senescent cells whether senescence was induced by doxorubicin (Figure 3C-i), irradiation (Figure 3C-ii) or etoposide (Figure 3C-iii) at statistically higher levels compared to cytotoxicity towards non-senescent cells (15%). In addition, the senescence phenotype may also vary depending on cell type. Thus the efficacy of enriched NK cells in targeting senescent endothelial cells was also investigated [38]. Enriched and activated NK cells were observed to be effective at killing senescent endothelial cells at a three-fold higher rate than NS endothelial cells (Figure 3C-iv) in a statistically significant manner.

Freezing primary NK cells often results in a decline in cytotoxicity [39]. However, our results showed that NK cells isolated and enriched with the protocol described above remained significantly more cytotoxic towards senescent cells compared to non-senescent cells following cryopreservation at a rate comparable to freshly isolated NK cells, as measured by LDH release after 16 hours of co-culture (Supplementary Figure 3B). Interestingly, when co-culture duration was extended to four days, the vast majority (85-90%) of NS cells survived, and only 10% and 30% of senescent cells were viable following co-culture with fresh or revived NK cells, respectively, as determined by Calcein AM (Supplementary Figure 3C).

Results from four days of co-culture were confirmed, demonstrating that only 6% and 17% of senescent cells survived following co-culture with NK cells even when senescence was induced by irradiation (Supplementary Figure 3D-i) or etoposide (Supplementary Figure 3D-ii), respectively, whereas 85-100% of non-senescent cells survived following co-culture.

Finally, to test whether the cytotoxicity of primary NK cells prepared using our protocol was influenced by donor variability, senescent or non-senescent IMR-90 cells were co-cultured with NK cells isolated from multiple donors. A significantly higher cytotoxicity towards senescent compared to non-senescent cells was observed, as measured by LDH assay for each donor (Figure 3D). Furthermore, although the levels of Granzyme B released by activated NK cells correlated with overall cytotoxicity efficiency of individual NK cell donors, NK cells from each donor released similar amounts of Granzyme B upon co-culture with either senescent or non-senescent cells (Supplementary Figure 3E).

## DISCUSSION

The involvement of senescent cells in aging and diseases of aging is well documented and has generated tremendous excitement amongst geroscientists as a potential therapeutic target for various age-related diseases. Several laboratories are attempting to develop therapeutic interventions to eliminate senescent cells using senolytic drug therapies. Although promising in animal models, unintended outcomes from senolytic interventions may be a potential concern [40–42]. A better understanding of the immune surveillance of senescent cells, and especially the key role of NK cells, offers a potential avenue to novel immunotherapies that can target senescent cells and expand healthspan.

NK cells are known to be one of the main effectors responsible for the immune surveillance of senescent cells. Hence, utilization of engineered NK cells as novel senotheraputics has emerged as an alternative to drug-based senolytic approaches. We investigated the viability of such an approach by demonstrating that our protocol is efficient in the isolation and enrichment of NK cells from PBMCs. Furthermore, this method can be used to assess the selective killing of senescent cells by NK cells in a practical and highly reproducible manner. Since senescence phenotype varies depending on the mode of senescence induction or cell type, we further showed that NK cells isolated and enriched with our protocol were also effective in killing senescent cells independent of cell origin or mode of senescence induction by doxorubicin, ionizing radiation or etoposide treatment.

To improve the efficiency of NK cell isolation, we isolated NK cells from PBMCs before expanding them in cell culture. The relative expression of CD56 and CD16 are commonly used to identify human NK cells [33]. CD56^dim^ (CD16^+^) cells are the predominant NK cell population in circulation, whereas CD56^bright^ (that are CD16^-^) cells are considered a less mature stage of NK cells [33, 43, 44]. Cytotoxic function is thought to be performed by CD56^dim^ NK cells, while CD56^bright^ NK cells are potent producers of cytokines such as IFN-γ and TNF-α [45, 46]. However, CD56^bright^ NK cells are known to acquire cytotoxic function in the presence of cytokines such as IL-2 [47]. Consistent with previous reports, we observed a higher proportion of CD56^dim^ NK cells in freshly isolated PBMCs. Following isolation and enrichment, we observed expansion of both CD56^dim^ and CD56^bright^ NK cell numbers by 15- and 3-fold, respectively, expressed as the mean of all five subjects at a relatively low rIL-2 concentration. IL-2 is considered essential for the proliferation and function of NK cells. Although other cytokines such as IL-4, IL-7, and IL-12 have been used for NK cell enrichment, they have been reported to be overall less potent [48]. Of note, cryopreserved NK cells that have been untouched by cytokines in cell culture have been shown to have phenotype and cytotoxicity that resembles those of fresh cells; whether this is true for IL-2 activated and expanded NK cells has not been established [39]. Interestingly, our data reveal that cryopreservation of IL-2 activated NK cells isolated and enriched via our method have comparable cytotoxicity to freshly isolated NK cells.

Our results show that NK cells kill 40-50% of senescent cells after 16 h at a T:E ratio of 1:1, which is more efficient and under more physiological conditions than has been previously reported. For instance, others have reported T:E ratios as high as 1:81 and co-culture durations as short as 2 h, which may not reflect physiological conditions [17, 18, 49]. A longer co-culture can also be useful for capturing the mechanism of Natural Killer Cell Cytotoxicity (NKCC). For example, in addition to releasing lytic granules to kill their targets within a few hours after the start of co-culture, NK cells are known to use additional mechanisms that require longer exposure to their targets, such as receptor-mediated apoptosis by expressing TRAIL or Fas ligand [50]. Thus, our improved co-culture strategy that employs more physiological T:E ratios (1:1) and longer co-culture durations (16 h or 4 d) likely captures broader and important NK-senescent cell interactions that may be more informative for studying the immune surveillance of senescent cells.

To test the robustness of our NK cell enrichment protocol, we isolated NK cells from five healthy individuals between the ages of 20-42 years. Our results show two- to three-fold higher cytotoxicity toward senescent cells when compared to non-senescent controls. Interestingly, our data suggest that NK cells from younger males generally exhibited higher cytotoxicity than females and older donors. This observation warrants further investigation with a larger sample size. Several studies have characterized changes in the numbers of circulating NK cells as well as the distribution of NK cell subsets with increasing age [51, 52]. Additionally, an age-related increase has been observed in the numbers of dysfunctional or exhausted NK cells, which can be identified by decreased NK effector functions, reduced IFN-γ secretion, and lower perforin and granzyme expression [53]. These observations a potential decline in NKCC with age. Whether this general age-related decline in NKCC contributes to the age-dependent increase in senescence burden in older adults should be investigated.

A greater understanding of the mechanisms by which NK cells interact with senescent cells is needed to identify novel interventions for improving immune surveillance by NK cells. Senescent cells are known to secrete CCL2, CCL5, CXCL9 and CXCL11 along with other SASP factors [54–57]. To confirm that our senescent cells secreted elevated levels of cytokines and chemoattractants, we tested gene expression levels of CCL2, CCL5, CXCL9 and CXCL11. We observed substantially higher expression of these cytokines from senescent compared to non-senescent cells, which is important for the context of future *in vivo* studies as these chemokines are known to be main chemoattractants for NK cells [58–60]. However, we did not observe an increase in the expression of CXCL12 or chemerin, which were reported to be critical chemotactic factors for NK cell recruitment, by senescent fibroblasts. Chemerin/RARRES2 is commonly downregulated across several tumor types and often employed by tumor cells to escape immune clearance by tumor-infiltrating effector leukocytes [61]. Our data suggest that senescent cells may also employ similar strategies *in vivo* to escape immune clearance. Since ectopic expression of chemerin in the tumor microenvironment (TME) results in increased recruitment of NK cells [62], a similar strategy could be used for improving immune surveillance of senescent cells by NK cells.

In summary, we have developed a robust, easily adaptable cell co-culture model that involves isolation of human NK cells from peripheral blood, followed by enrichment in a relatively low IL-2 concentration. Our method for co-culturing NK cells with senescent cells may more fully capture the highly specific cytotoxicity towards senescent human fibroblasts and endothelial cells under more physiological conditions. We achieved significant cytotoxicity of NK cells towards senescent cells independent of donor variability using our enrichment strategy. Moreover, the specificity of primary NK cells towards senescent cells was further confirmed by a 4-day co-culture in which virtually all senescent cells were killed whereas viability of non-senescent cells with NK cell effectors was visually indistinguishable from negative control cells untouched by effector cells. A deeper understanding of the interplay between senescent cells and NK cells is essential for the development of better therapeutic interventions, especially novel immunotherapies, for the treatment and prevention of age-associated diseases. The co-culture strategy presented here may serve as an important platform for the development of effective immunotherapies in the future that may involve the alteration and ultimately improvement of NK cell cytotoxicity towards senescent cells.

## MATERIALS AND METHODS

### Cell culture

Human primary NK cells were maintained at 37° C in humidified air containing 5% CO_2_, and complete media containing RPMI-1640 medium (ATCC, USA; Cat#30-2001) supplemented with 20% Fetal Bovine Serum (FBS) (Millipore Sigma, USA; Cat# F4135), 1X Penicillin–Streptomycin (Corning; Cat# 30-001-CI), and 100 IU/ml human rIL-2 (recombinant Interleukin-2) (TECIN teceleukin; Bulk Ro 23-6019). IMR-90 primary lung fibroblasts (ATCC, USA: Cat# CCL-186) were used at population doubling level (PDL) 30-47 and were cultured in an atmosphere with 5% CO_2_ and 3% O_2_ with complete media containing Dulbecco’s Modified Eagle’s Medium (DMEM) (Corning; Cat# 10-013-CV) supplemented with 10% Fetal Bovine Serum (FBS) (Millipore Sigma, US; Cat# F4135) and 1X Penicillin–Streptomycin (Corning; Cat# 30-001-CI). Cumulative PDL was calculated using the following equation:


$$\text{PDL}=\frac{\text{log}\;\text{H}-\text{log}\;\text{S}}{\text{log}\;\text{2}}$$

where H is the number of cells at harvest and S is the number of cells seeded. Primary human arterial endothelial cells purchased from Coriell Institute for medical research (AG10770) were maintained in promo cell basal medium MV2 (PromoCell; Cat# C-22221) supplemented with Growth Medium MV 2 Supplement Pack (PromoCell; Cat# C-39221) and assayed within 10 or less passages. Endothelial cells were maintained at 37° C in humidified air containing 5% CO_2_.

### Senescence induction

Senescence was induced in IMR-90 fibroblasts as described before with some modifications [63]. Cells were treated with 300 nM doxorubicin hydrochloride (Millipore Sigma, USA; Cat# 504042) in DMEM complete media for 24 h and maintained in culture as described for 10 days (d). Human endothelial cells were treated with 250 nM doxorubicin in promo cell basal medium MV2 supplemented with Growth Medium MV 2 Supplement Pack for 24 h and maintained in culture as described [63]. For cells induced to senesce by irradiation, IMR-90 cells were treated with ionizing radiation (20 Gy X-ray). For cells induced to senesce by etoposide, fibroblasts were treated with 20 μM of etoposide (Millipore Sigma, USA; Cat# E1383) for 48 h and maintained in culture as described before.

### Senescence-associated ß-galactosidase staining

Senescence in IMR-90 cells was determined by senescence-associated ß-galactosidase (SA-ß-gal) activity, as reported [32], using the Senescence Detection Kit (BioVision; Cat# K320), following the manufacturer’s instructions. Fibroblasts were plated (5 x 10^4^ per well) 1 day before treatment in a 6-well cell culture plate (Greiner Bio-One; Cat# 657160) with 2.5 ml of DMEM complete media per well. Non-senescent cells were plated (1 x 10^5^) also in 6-well cell culture plates 1 d before staining. Staining was performed 9 d after doxorubicin treatment. During staining, cells were incubated for 48 h at 37° C in the absence of CO_2_, then visualized by bright-field microscopy and imaged. Percentage of SA-ß-gal-positive cells was counted manually.

### Real-time quantitative PCR

Non-senescent IMR-90 fibroblasts were plated (1 x 10^6^) in a T-75 cell culture flask (Cellstar; Cat# 658170) with 10 ml of DMEM complete media 2 d before cell pellet harvest. For senescent cells, fibroblasts were plated (1 x 10^6^) 1 d before doxorubicin treatment also in a T-75 cell culture flask, with 10 ml of DMEM complete media. Senescent cells were pelleted 9 d after doxorubicin treatment. All cell pellets were stored at -80° C before RNA isolation. Total RNA was isolated from cell pellets using Quick-RNA MiniPrep (Zymo Research; Cat# R1055), following the manufacturer’s protocol. 1 μg of total RNA per sample was reverse transcribed using PrimeScript RT Master Mix (Takara; Cat# RR036B), and cDNA was analyzed by real-time qPCR using TaqMan Fast Advanced Master Mix (Applied Biosystems; Cat# 4444557) (StepOnePlus™ Real-Time PCR System). Gene expression analyses were performed with Applied Biosystems TaqMan Gene Expression single-tube assays. All reactions were performed in triplicate, and relative expression levels of each gene were normalized to actin. The relative expression of mRNA was determined using the comparative threshold (Ct) method by normalizing target cDNA Ct values to that of actin.

### Immunofluorescence (IF)

IMR-90 fibroblasts were plated for senescence induction (2 x 10^4^/well) 1 d before doxorubicin treatment in a black 96-well plate with square wells and a flat, clear bottom (Ibidi; Cat# 89626), with 250 μl/well of DMEM complete media. Non-senescent cells were plated (2 x 10^5/well) 2 d before staining. All immunostaining was performed 8 d after doxorubicin treatment. Cells were fixed with 200 μl/well of 4% paraformaldehyde in 1X PBS (Thermo Scientific; Cat# AAJ19943K2) for 15 min at room temperature, carefully rinsed with 1X PBS (Corning; Cat# 21-031-CV), then permeabilized with 300 μl per well of 0.5% Triton X-100 for 10 min at room temperature. Cells were rinsed once with 1X PBS and incubated with 250 μl per well of anti-γH2AX [p Ser139] (Novus Biologicals; Cat# NB100-74435) and anti-HMGB1 (abcam; Cat# ab18256) antibodies diluted in 5% BSA (Research Products International; Cat# A30075) in 1X PBS overnight at 4° C. Subsequently, cells were washed 5 times with 1X PBS and incubated with 250 μl/well of both Alexa Fluor 488 goat anti-mouse antibody (Invitrogen; Cat# A11029) and Alexa Fluor 546 goat anti-rabbit antibody (Invitrogen; Cat# A11010) along with Hoechst 33342, trihydrochloride, trihydrate (Invitrogen; Cat# H3570) in 5% BSA for 20 min at room temperature in the dark. Cells were washed 5 times with 1X PBS, then 200 μl of 1X PBS was added to each well before images were acquired with Molecular Devices Image Express Micro (Molecular Devices, San Jose, CA, USA). Seven images were acquired per well, with five wells captured per condition (NS or S). Cells with >2 γ-H2AX foci per nucleus were defined as senescent (S).

### NK cell isolation and enrichment

Blood samples were obtained from healthy donors (n = 5, age range 20-42) in heparin coated vacutainers. All subjects provided informed written consent. Inclusion criteria for healthy individuals included those who did not take medication that could impact immunity (e.g., corticosteroids) and had no clinical indication of immunodeficiency. NK cells were isolated from freshly drawn human peripheral blood with RosetteSep Human NK Cell Enrichment Cocktail (Stemcell Technology, USA; Cat# 15065) 3 d before co-culture with target cells. Rosette antibody cocktail was added to each blood sample (30 μl/ml) to negatively select unwanted cells with Tetrameric Antibody Complexes that crosslink non-NK cells in human whole blood to red blood cells (RBCs). Blood samples were then incubated at room temperature for 40 min, diluted with 1X PBS (Corning; Cat# 21-031-CV), and combined with Lymphocyte Separation Medium (Corning; Cat# 25-072-CI) followed by density gradient centrifugation, according to the manufacturer’s instructions. Once isolated, NK cells were enriched and activated for 3 days in RPMI complete media with 100 IU/ml human rIL-2 (recombinant Interleukin-2) (R and D Systems; Cat# 8879-IL-010) before co-culturing with senescent or non-senescent IMR-90 fibroblasts.

### NK cell characterization

Peripheral blood mononuclear cells (PBMCs) or enriched NK cells were resuspended to a concentration of 1 x 10^6^ cells/ml in FACS buffer (1% BSA in PBS + 0.01% sodium azide) and aliquoted. Cells were washed 2 times with 1X PBS and resuspended in FACS buffer. Cells were then incubated with APC-conjugated anti-human CD3 antibody (Miltenyi Biotec; Cat# 130-113-135), PE-conjugated anti-human CD16 antibody (Miltenyi Biotec; Cat# 130-113-393) and FITC-conjugated anti-human CD56 antibody (Miltenyi Biotec; Cat# 130-114-549) for 30 min at room temperature in the dark. Then, cells were washed 3 times with FACS buffer and resuspended in 200 μl of FACS buffer. Finally, 500,000 events were collected by flow cytometer (DB Accuri C6). Cell viability was determined by PI staining and live cells were gated for downstream analysis. Lymphocytes were gated based on forward and side scatter followed by gating to CD3- cells as NK cells. Finally, percentages of CD56dim CD16+ and CD56bright CD16- NK cells were determined across multiple donors. The data were acquired for at least 100,000 cells/sample. Data were analyzed using Flowlogic software (Miltenyi Biotech, Germany).

### Method for co-culture of NK effector cells with IMR-90 target cells

IMR-90 fibroblasts were seeded (2 x 10^4^/well) onto 24-well cell culture plates (Greiner Bio-One; Cat# 662160) with a 1 ml volume of DMEM complete media 10 d before co-culture. Senescence was induced 9 d before co-culture by treatment with 300 nM of doxorubicin for 24 h. Media was replaced with fresh DMEM complete media every 2-3 d. 3 d before co-culture with NK cells, non-senescent IMR-90 fibroblasts were seeded (1 x 10^4 per well) onto 24-well cell culture plates (Greiner Bio-One; Cat# 662160) with 1 ml DMEM complete media. 24 h before co-culture, each well was replaced with 250 μl/well of DMEM complete media to allow for SASP accumulation from senescent cells. Additional wells containing no target cells were included for media only and NK cell spontaneous lactate dehydrogenase (LDH) release controls. At the start of co-culture, NK cells were added in 250μl/well of RPMI complete media at a target to effector (T:E) ratio of 1:1 for a total of volume of 500 μl per well. Co-cultures were incubated at 37° C in humidified air containing 5% CO_2_ for 16 h. Bright-field images were taken after 16 h of co-culture for qualitative analysis of NK cell cytotoxicity.

### Cytotoxicity quantification

Cytotoxicity was assessed after 16 h of co-culture incubation by quantifying LDH release levels in target cells using the CytoTox 96 Non-Radioactive Cytotoxicity Assay (Promega, Madison, WI, USA), which quantitatively measures LDH released upon cell lysis. Baseline expression of LDH in culture medium alone, medium with target cells only, and medium with NK cells only were used as negative controls. Fibroblasts treated with 0.2% Triton X-100 served as positive controls. Absorbance was recorded at 490 nm using SpectraMax i3 Multi-Mode Microplate Reader (Molecular Devices, San Jose, CA, USA). Average absorbance values for the culture medium background were subtracted from absorbance values for experimental and target cell spontaneous LDH release. The data are presented as an index calculated as:


$$\%\;\;Cytotoxicity=\frac{\begin{array}{c} Experimental-Effector\;\\ Spontaneous\\ -Target\;Spontaneous \end{array}}{\begin{array}{c} Positive\;control\\ -Target\;Spontaneous \end{array}}\;\times \;100$$

### Granzyme B release assay

Secretion of granzyme B was determined in the supernatant of NK/IMR90-cell co-cultures after 16 h. Co-culture plates were subject to centrifugation at 400xg for 5 min. Supernatant was collected and used to measure human granzyme B by enzyme-linked immunosorbent assay (ELISA) kit (R and D Systems; DY2906-05), according to the manufacturer’s instructions.

### Cell viability assay with Calcein AM

Fibroblasts were carefully rinsed 4 times with 1X PBS (1 ml/well) to wash away effector (NK cells) and dead detached cells. Target cells were stained with 1 μM of Calcein AM (diluted from a 4 mM stock solution in dimethyl sulfoxide (DMSO), Invitrogen; Cat# C3099) in no-serum DMEM for 30 min in a 37° C incubator at a volume of 250 μl/well. Following incubation, fluorescence values were recorded at 530 nm using SpectraMax i3 Multi-Mode Microplate Reader (Molecular Devices, San Jose, CA, USA).

## Supplementary Material

Supplementary Figures

## References

[1] Rae MJ, Butler RN, Campisi J, de Grey AD, Finch CE, Gough M, Martin GM, Vijg J, Perrott KM, Logan BJ. The demographic and biomedical case for late-life interventions in aging. Sci Transl Med. 2010; 2:40cm21. 10.1126/scitranslmed.3000822 20630854 PMC3677970

[2] Espeland MA, Crimmins EM, Grossardt BR, Crandall JP, Gelfond JA, Harris TB, Kritchevsky SB, Manson JE, Robinson JG, Rocca WA, Temprosa M, Thomas F, Wallace R, Barzilai N, and Multimorbidity Clinical Trials Consortium. Clinical Trials Targeting Aging and Age-Related Multimorbidity. J Gerontol A Biol Sci Med Sci. 2017; 72:355–61. 10.1093/gerona/glw220 28364543 PMC5777384

[3] Baker DJ, Wijshake T, Tchkonia T, LeBrasseur NK, Childs BG, van de Sluis B, Kirkland JL, van Deursen JM. Clearance of p16Ink4a-positive senescent cells delays ageing-associated disorders. Nature. 2011; 479:232–6. 10.1038/nature10600 22048312 PMC3468323

[4] Ghosh K, Capell BC. The Senescence-Associated Secretory Phenotype: Critical Effector in Skin Cancer and Aging. J Invest Dermatol. 2016; 136:2133–9. 10.1016/j.jid.2016.06.621 27543988 PMC5526201

[5] Zhang ZD, Milman S, Lin JR, Wierbowski S, Yu H, Barzilai N, Gorbunova V, Ladiges WC, Niedernhofer LJ, Suh Y, Robbins PD, Vijg J. Genetics of extreme human longevity to guide drug discovery for healthy ageing. Nat Metab. 2020; 2:663–72. 10.1038/s42255-020-0247-0 32719537 PMC7912776

[6] Prata LG, Ovsyannikova IG, Tchkonia T, Kirkland JL. Senescent cell clearance by the immune system: emerging therapeutic opportunities. Semin Immunol. 2018; 40:101275. 10.1016/j.smim.2019.04.003 31088710 PMC7061456

[7] Kirkland JL, Tchkonia T. Clinical strategies and animal models for developing senolytic agents. Exp Gerontol. 2015; 68:19–25. 10.1016/j.exger.2014.10.012 25446976 PMC4412760

[8] Baker DJ, Childs BG, Durik M, Wijers ME, Sieben CJ, Zhong J, Saltness RA, Jeganathan KB, Verzosa GC, Pezeshki A, Khazaie K, Miller JD, van Deursen JM. Naturally occurring p16(Ink4a)-positive cells shorten healthy lifespan. Nature. 2016; 530:184–9. 10.1038/nature16932 26840489 PMC4845101

[9] Kale A, Sharma A, Stolzing A, Desprez PY, Campisi J. Role of immune cells in the removal of deleterious senescent cells. Immun Ageing. 2020; 17:16. 10.1186/s12979-020-00187-9 32518575 PMC7271494

[10] Hoenicke L, Zender L. Immune surveillance of senescent cells—biological significance in cancer- and non-cancer pathologies. Carcinogenesis. 2012; 33:1123–6. 10.1093/carcin/bgs124 22470164

[11] Sagiv A, Biran A, Yon M, Simon J, Lowe SW, Krizhanovsky V. Granule exocytosis mediates immune surveillance of senescent cells. Oncogene. 2013; 32:1971–7. 10.1038/onc.2012.206 22751116 PMC3630483

[12] Bataller R, Brenner DA. Liver fibrosis. J Clin Invest. 2005; 115:209–18. 10.1172/JCI24282 15690074 PMC546435

[13] Brighton PJ, Maruyama Y, Fishwick K, Vrljicak P, Tewary S, Fujihara R, Muter J, Lucas ES, Yamada T, Woods L, Lucciola R, Hou Lee Y, Takeda S, et al. Clearance of senescent decidual cells by uterine natural killer cells in cycling human endometrium. eLife. 2017; 6:e31274. 10.7554/eLife.31274 29227245 PMC5724991

[14] Xue W, Zender L, Miething C, Dickins RA, Hernando E, Krizhanovsky V, Cordon-Cardo C, Lowe SW. Senescence and tumour clearance is triggered by p53 restoration in murine liver carcinomas. Nature. 2007; 445:656–60. 10.1038/nature05529 17251933 PMC4601097

[15] Ovadya Y, Landsberger T, Leins H, Vadai E, Gal H, Biran A, Yosef R, Sagiv A, Agrawal A, Shapira A, Windheim J, Tsoory M, Schirmbeck R, et al. Impaired immune surveillance accelerates accumulation of senescent cells and aging. Nat Commun. 2018; 9:5435. 10.1038/s41467-018-07825-3 30575733 PMC6303397

[16] Bernardini G, Gismondi A, Santoni A. Chemokines and NK cells: regulators of development, trafficking and functions. Immunol Lett. 2012; 145:39–46. 10.1016/j.imlet.2012.04.014 22698182 PMC7112821

[17] Pereira BI, Devine OP, Vukmanovic-Stejic M, Chambers ES, Subramanian P, Patel N, Virasami A, Sebire NJ, Kinsler V, Valdovinos A, LeSaux CJ, Passos JF, Antoniou A, et al. Senescent cells evade immune clearance via HLA-E-mediated NK and CD8^+^ T cell inhibition. Nat Commun. 2019; 10:2387. 10.1038/s41467-019-10335-5 31160572 PMC6547655

[18] Muñoz DP, Yannone SM, Daemen A, Sun Y, Vakar-Lopez F, Kawahara M, Freund AM, Rodier F, Wu JD, Desprez PY, Raulet DH, Nelson PS, van ’t Veer LJ, et al. Targetable mechanisms driving immunoevasion of persistent senescent cells link chemotherapy-resistant cancer to aging. JCI Insight. 2019; 5:e124716. 10.1172/jci.insight.124716 31184599 PMC6675550

[19] Zingoni A, Cecere F, Vulpis E, Fionda C, Molfetta R, Soriani A, Petrucci MT, Ricciardi MR, Fuerst D, Amendola MG, Mytilineos J, Cerboni C, Paolini R, et al. Genotoxic Stress Induces Senescence-Associated ADAM10-Dependent Release of NKG2D MIC Ligands in Multiple Myeloma Cells. J Immunol. 2015; 195:736–48. 10.4049/jimmunol.1402643 26071561

[20] Choi PJ, Mitchison TJ. Imaging burst kinetics and spatial coordination during serial killing by single natural killer cells. Proc Natl Acad Sci USA. 2013; 110:6488–93. 10.1073/pnas.1221312110 23576740 PMC3631668

[21] Vanherberghen B, Olofsson PE, Forslund E, Sternberg-Simon M, Khorshidi MA, Pacouret S, Guldevall K, Enqvist M, Malmberg KJ, Mehr R, Önfelt B. Classification of human natural killer cells based on migration behavior and cytotoxic response. Blood. 2013; 121:1326–34. 10.1182/blood-2012-06-439851 23287857

[22] Perera Molligoda Arachchige AS. Human NK cells: from development to effector functions. Innate Immun. 2021; 27:212–29. 10.1177/17534259211001512 33761782 PMC8054151

[23] Krizhanovsky V, Yon M, Dickins RA, Hearn S, Simon J, Miething C, Yee H, Zender L, Lowe SW. Senescence of activated stellate cells limits liver fibrosis. Cell. 2008; 134:657–67. 10.1016/j.cell.2008.06.049 18724938 PMC3073300

[24] Ruscetti M, Leibold J, Bott MJ, Fennell M, Kulick A, Salgado NR, Chen CC, Ho YJ, Sanchez-Rivera FJ, Feucht J, Baslan T, Tian S, Chen HA, et al. NK cell-mediated cytotoxicity contributes to tumor control by a cytostatic drug combination. Science. 2018; 362:1416–22. 10.1126/science.aas9090 30573629 PMC6711172

[25] Iannello A, Thompson TW, Ardolino M, Lowe SW, Raulet DH. p53-dependent chemokine production by senescent tumor cells supports NKG2D-dependent tumor elimination by natural killer cells. J Exp Med. 2013; 210:2057–69. 10.1084/jem.20130783 24043758 PMC3782044

[26] Weil-Hillman G, Voss SD, Fisch P, Schell K, Hank JA, Sosman JA, Sugamura K, Sondel PM. Natural killer cells activated by interleukin 2 treatment *in vivo* respond to interleukin 2 primarily through the p75 receptor and maintain the p55 (TAC) negative phenotype. Cancer Res. 1990; 50:2683–91. 1691679

[27] Yin X, Xu X, Zhao Y, Wang ZJ, Wang HY, Hu ZB. [Comparison of Several Optimization Schemes for the Induction and Expansion of Antibody-Mediated High Efficiency CIK (AMHE-CIK) *In Vitro*]. Zhongguo Shi Yan Xue Ye Xue Za Zhi. 2016; 24:191–6. 10.7534/j.issn.1009-2137.2016.01.036 26913419

[28] Wei C, Wang W, Pang W, Meng M, Jiang L, Xue S, Xie Y, Li R, Hou Z. The CIK cells stimulated with combination of IL-2 and IL-15 provide an improved cytotoxic capacity against human lung adenocarcinoma. Tumour Biol. 2014; 35:1997–2007. 10.1007/s13277-013-1265-2 24104501

[29] Davalos AR, Kawahara M, Malhotra GK, Schaum N, Huang J, Ved U, Beausejour CM, Coppe JP, Rodier F, Campisi J. p53-dependent release of Alarmin HMGB1 is a central mediator of senescent phenotypes. J Cell Biol. 2013; 201:613–29. 10.1083/jcb.201206006 23649808 PMC3653366

[30] Freund A, Laberge RM, Demaria M, Campisi J. Lamin B1 loss is a senescence-associated biomarker. Mol Biol Cell. 2012; 23:2066–75. 10.1091/mbc.e11-10-0884 22496421 PMC3364172

[31] Coppé JP, Patil CK, Rodier F, Krtolica A, Beauséjour CM, Parrinello S, Hodgson JG, Chin K, Desprez PY, Campisi J. A human-like senescence-associated secretory phenotype is conserved in mouse cells dependent on physiological oxygen. PLoS One. 2010; 5:e9188. 10.1371/journal.pone.0009188 20169192 PMC2820538

[32] Laberge RM, Sun Y, Orjalo AV, Patil CK, Freund A, Zhou L, Curran SC, Davalos AR, Wilson-Edell KA, Liu S, Limbad C, Demaria M, Li P, et al. MTOR regulates the pro-tumorigenic senescence-associated secretory phenotype by promoting IL1A translation. Nat Cell Biol. 2015; 17:1049–61. 10.1038/ncb3195 26147250 PMC4691706

[33] Poli A, Michel T, Thérésine M, Andrès E, Hentges F, Zimmer J. CD56bright natural killer (NK) cells: an important NK cell subset. Immunology. 2009; 126:458–65. 10.1111/j.1365-2567.2008.03027.x 19278419 PMC2673358

[34] Moretta A, Bottino C, Vitale M, Pende D, Cantoni C, Mingari MC, Biassoni R, Moretta L. Activating receptors and coreceptors involved in human natural killer cell-mediated cytolysis. Annu Rev Immunol. 2001; 19:197–223. 10.1146/annurev.immunol.19.1.197 11244035

[35] Voskoboinik I, Smyth MJ, Trapani JA. Perforin-mediated target-cell death and immune homeostasis. Nat Rev Immunol. 2006; 6:940–52. 10.1038/nri1983 17124515

[36] Antonangeli F, Zingoni A, Soriani A, Santoni A. Senescent cells: living or dying is a matter of NK cells. J Leukoc Biol. 2019; 105:1275–83. 10.1002/JLB.MR0718-299R 30811627

[37] Admasu TD, Rae M, Stolzing A. Dissecting primary and secondary senescence to enable new senotherapeutic strategies. Ageing Res Rev. 2021; 70:101412. 10.1016/j.arr.2021.101412 34302996

[38] Salama R, Sadaie M, Hoare M, Narita M. Cellular senescence and its effector programs. Genes Dev. 2014; 28:99–114. 10.1101/gad.235184.113 24449267 PMC3909793

[39] Cho D, Campana D. Expansion and activation of natural killer cells for cancer immunotherapy. Korean J Lab Med. 2009; 29:89–96. 10.3343/kjlm.2009.29.2.89 19411773 PMC2771620

[40] Blagosklonny MV. Paradoxes of senolytics. Aging (Albany NY). 2018; 10:4289–93. 10.18632/aging.101750 30594910 PMC6326665

[41] Kirkland JL, Tchkonia T. Cellular Senescence: A Translational Perspective. EBioMedicine. 2017; 21:21–8. 10.1016/j.ebiom.2017.04.013 28416161 PMC5514381

[42] Zhu Y, Tchkonia T, Fuhrmann-Stroissnigg H, Dai HM, Ling YY, Stout MB, Pirtskhalava T, Giorgadze N, Johnson KO, Giles CB, Wren JD, Niedernhofer LJ, Robbins PD, Kirkland JL. Identification of a novel senolytic agent, navitoclax, targeting the Bcl-2 family of anti-apoptotic factors. Aging Cell. 2016; 15:428–35. 10.1111/acel.12445 26711051 PMC4854923

[43] Carrega P, Bonaccorsi I, Di Carlo E, Morandi B, Paul P, Rizzello V, Cipollone G, Navarra G, Mingari MC, Moretta L, Ferlazzo G. CD56(bright)perforin(low) noncytotoxic human NK cells are abundant in both healthy and neoplastic solid tissues and recirculate to secondary lymphoid organs via afferent lymph. J Immunol. 2014; 192:3805–15. 10.4049/jimmunol.1301889 24646734

[44] Lugthart G, Melsen JE, Vervat C, van Ostaijen-Ten Dam MM, Corver WE, Roelen DL, van Bergen J, van Tol MJ, Lankester AC, Schilham MW. Human Lymphoid Tissues Harbor a Distinct CD69+CXCR6+ NK Cell Population. J Immunol. 2016; 197:78–84. 10.4049/jimmunol.1502603 27226093

[45] Michel T, Poli A, Cuapio A, Briquemont B, Iserentant G, Ollert M, Zimmer J. Human CD56bright NK Cells: an Update. J Immunol. 2016; 196:2923–31. 10.4049/jimmunol.1502570 26994304

[46] Penack O, Gentilini C, Fischer L, Asemissen AM, Scheibenbogen C, Thiel E, Uharek L. CD56dimCD16neg cells are responsible for natural cytotoxicity against tumor targets. Leukemia. 2005; 19:835–40. 10.1038/sj.leu.2403704 15744340

[47] Ferlazzo G, Thomas D, Lin SL, Goodman K, Morandi B, Muller WA, Moretta A, Münz C. The abundant NK cells in human secondary lymphoid tissues require activation to express killer cell Ig-like receptors and become cytolytic. J Immunol. 2004; 172:1455–62. 10.4049/jimmunol.172.3.1455 14734722

[48] Robertson MJ, Manley TJ, Donahue C, Levine H, Ritz J. Costimulatory signals are required for optimal proliferation of human natural killer cells. J Immunol. 1993; 150:1705–14. 7679691

[49] Sagiv A, Burton DG, Moshayev Z, Vadai E, Wensveen F, Ben-Dor S, Golani O, Polic B, Krizhanovsky V. NKG2D ligands mediate immunosurveillance of senescent cells. Aging (Albany NY). 2016; 8:328–44. 10.18632/aging.100897 26878797 PMC4789586

[50] Prager I, Watzl C. Mechanisms of natural killer cell-mediated cellular cytotoxicity. J Leukoc Biol. 2019; 105:1319–29. 10.1002/JLB.MR0718-269R 31107565

[51] Almeida-Oliveira A, Smith-Carvalho M, Porto LC, Cardoso-Oliveira J, Ribeiro AS, Falcão RR, Abdelhay E, Bouzas LF, Thuler LC, Ornellas MH, Diamond HR. Age-related changes in natural killer cell receptors from childhood through old age. Hum Immunol. 2011; 72:319–29. 10.1016/j.humimm.2011.01.009 21262312

[52] Chidrawar SM, Khan N, Chan YL, Nayak L, Moss PA. Ageing is associated with a decline in peripheral blood CD56bright NK cells. Immun Ageing. 2006; 3:10. 10.1186/1742-4933-3-10 17134511 PMC1702551

[53] Bi J, Tian Z. NK Cell Exhaustion. Front Immunol. 2017; 8:760. 10.3389/fimmu.2017.00760 28702032 PMC5487399

[54] Hwang HJ, Lee YR, Kang D, Lee HC, Seo HR, Ryu JK, Kim YN, Ko YG, Park HJ, Lee JS. Endothelial cells under therapy-induced senescence secrete CXCL11, which increases aggressiveness of breast cancer cells. Cancer Lett. 2020; 490:100–10. 10.1016/j.canlet.2020.06.019 32659248

[55] Yamane M, Sato S, Shimizu E, Shibata S, Hayano M, Yaguchi T, Kamijuku H, Ogawa M, Suzuki T, Mukai S, Shimmura S, Okano H, Takeuchi T, et al. Senescence-associated secretory phenotype promotes chronic ocular graft-vs-host disease in mice and humans. FASEB J. 2020; 34:10778–800. 10.1096/fj.201900218R 32619061

[56] Shen L, Chen Y, Cheng J, Yuan S, Zhou S, Yan W, Liu J, Luo A, Wang S. CCL5 secreted by senescent theca-interstitial cells inhibits preantral follicular development via granulosa cellular apoptosis. J Cell Physiol. 2019; 234:22554–64. 10.1002/jcp.28819 31111482

[57] Liu Y, Pan J, Pan X, Wu L, Bian J, Lin Z, Xue M, Su T, Lai S, Chen F, Ge Q, Chen L, Ye S, et al. Klotho-mediated targeting of CCL2 suppresses the induction of colorectal cancer progression by stromal cell senescent microenvironments. Mol Oncol. 2019; 13:2460–75. 10.1002/1878-0261.12577 31545552 PMC6822285

[58] Dabiri S, Kariminik A, Kennedy D. The role of CXCR3 and its ligands in renal transplant outcome. Eur Cytokine Netw. 2016; 27:34–40. 10.1684/ecn.2016.0375 27478077

[59] Bhat H, Zaun G, Hamdan TA, Lang J, Adomati T, Schmitz R, Friedrich SK, Bergerhausen M, Cham LB, Li F, Ali M, Zhou F, Khairnar V, et al. Arenavirus Induced CCL5 Expression Causes NK Cell-Mediated Melanoma Regression. Front Immunol. 2020; 11:1849. 10.3389/fimmu.2020.01849 32973762 PMC7472885

[60] Shou Q, Fu H, Huang X, Yang Y. PARP-1 controls NK cell recruitment to the site of viral infection. JCI Insight. 2019; 4:121291. 10.1172/jci.insight.121291 31217354 PMC6629106

[61] Shin WJ, Pachynski RK. Chemerin modulation of tumor growth: potential clinical applications in cancer. Discov Med. 2018; 26:31–7. 30265853

[62] Rennier K, Shin WJ, Krug E, Virdi G, Pachynski RK. Chemerin Reactivates PTEN and Suppresses PD-L1 in Tumor Cells via Modulation of a Novel CMKLR1-mediated Signaling Cascade. Clin Cancer Res. 2020; 26:5019–35. 10.1158/1078-0432.CCR-19-4245 32605911

[63] Demaria M, O’Leary MN, Chang J, Shao L, Liu S, Alimirah F, Koenig K, Le C, Mitin N, Deal AM, Alston S, Academia EC, Kilmarx S, et al. Cellular senescence promotes adverse effects of chemotherapy and cancer relapse. Cancer Discov. 2017; 7:165–76. 10.1158/2159-8290.CD-16-0241 27979832 PMC5296251

