# Supplementary material for: Enhanced co-culture and enrichment of human natural killer cells for the selective clearance of senescent cells
Source: Aging (Albany NY). 2022 Mar 4;14(5):2131–47. doi: 10.18632/aging.203931 (PMC8954966; doi:10.18632/aging.203931)
Supplement: Supplementary Figures [file aging-14-203931-s001.pdf]

## SUPPLEMENTARY FIGURES

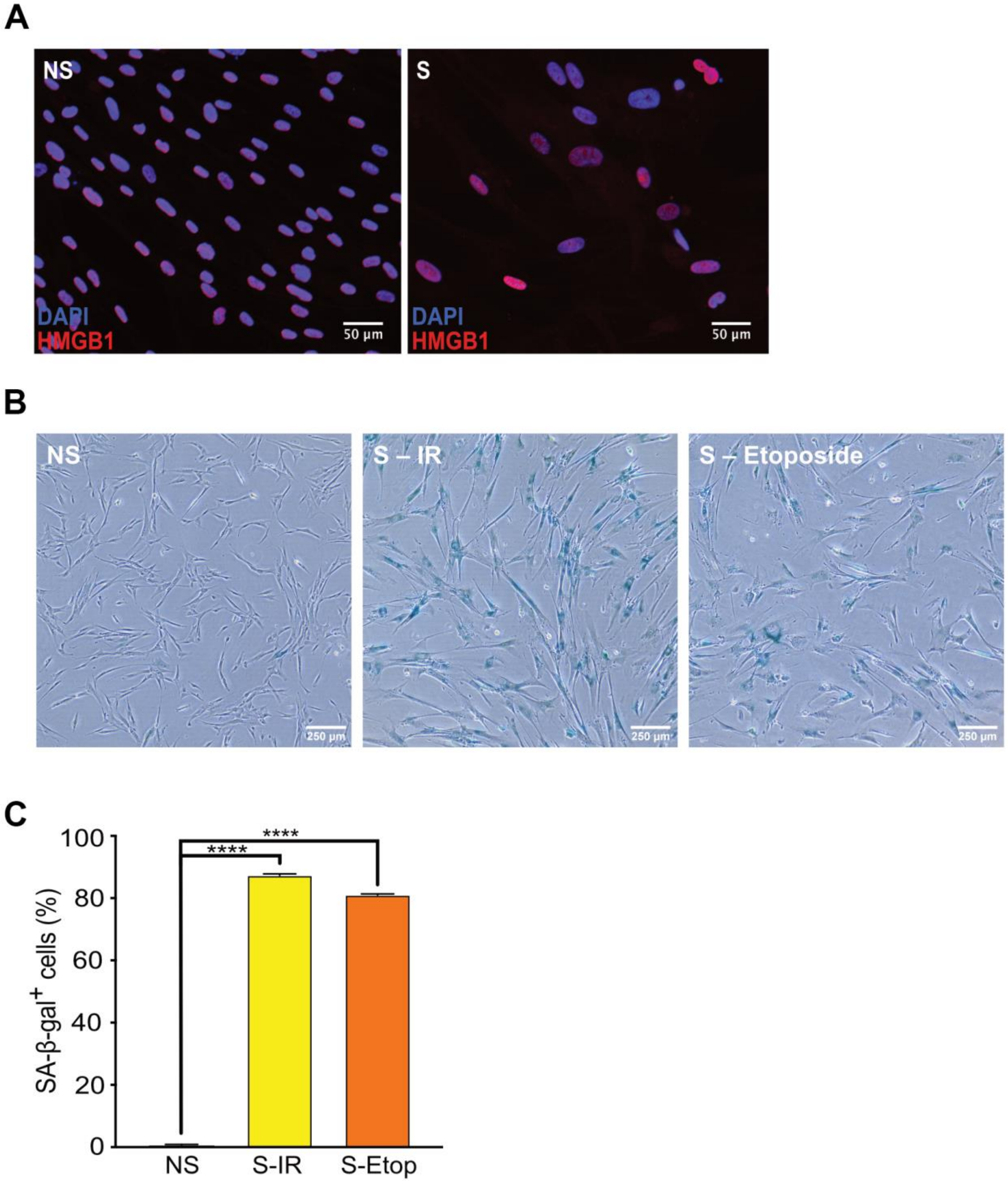


**Supplementary Figure 1. Senescent IMR-90 cells express markers of senescence.** Related to Figure 1. (**A**) Representative immunofluorescence images of NS and S IMR-90 cells stained for HMGB1 (red). Nuclei were stained with Hoechst (blue). (**B**) Representative images of SA-β-Gal-stained senescent (S – IR, S – Etoposide) and non-senescent (NS) IMR-90 cells. SA-β-Gal staining was performed on day 9 after treatment with irradiation (20 Gy) or Etoposide (20 μM, 48 h). (**C**) Quantification of SA-β-gal-positive IMR-90 cells in NS, S – IR and S – Etoposide. Four fields were quantified per well (n=3) with a total of 3483, 1079 and 777 cells counted for NS, S – IR and S – Etoposide, respectively.

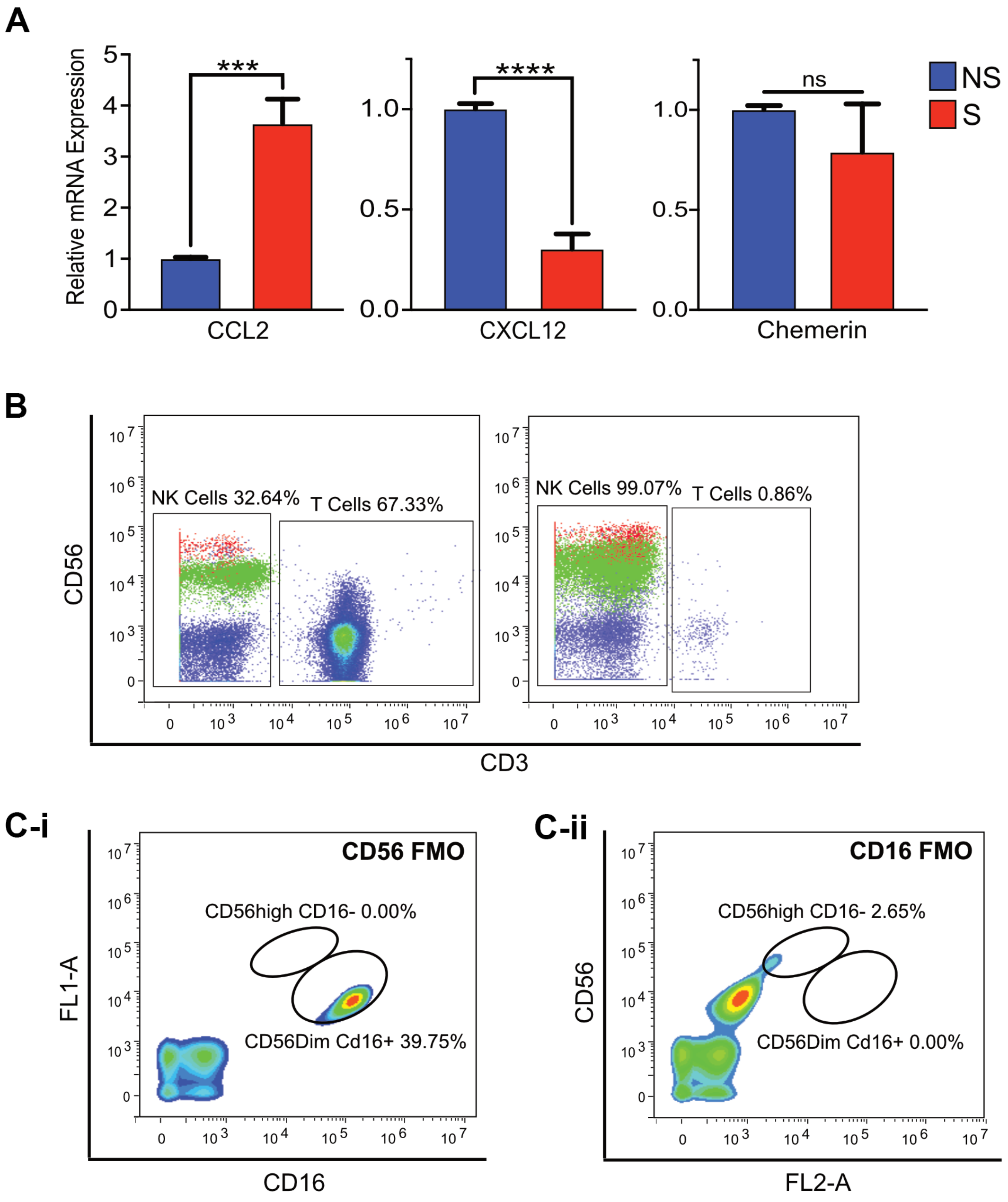


**Supplementary Figure 2. Cytokine expression of non-senescent versus senescent IMR-90 cells and NK cell enrichment.** (**A**) qRT-PCR of *CCL2*, *CXCL12* and *Chemerin* mRNA in non-senescent and senescent IMR-90 fibroblasts. The results are presented as mean fold change in NS compared to S of two independent experiments performed in triplicate, and error bars represent ±SEM. Statistical analysis performed using unpaired t test. *p < 0.05, **p < 0.01, and ***p < 0.001. (**B**) Flow cytometry analysis of CD56 and CD3 expressing cells before (left) and after (right) enrichment. Rosette antibody-based isolation resulted in a pure population (99.07%) of NK cells. Red is $CD56^{bright}$ and green is $CD56^{dim}$ NK cells. (**C-i**) Fluorescence Minus One (FMO) control for CD56 from a representative donor. NK cell populations were stained with anti-CD16 antibody. (**C-ii**) FMO control for CD16. NK cell populations were stained with anti-CD56 antibody.

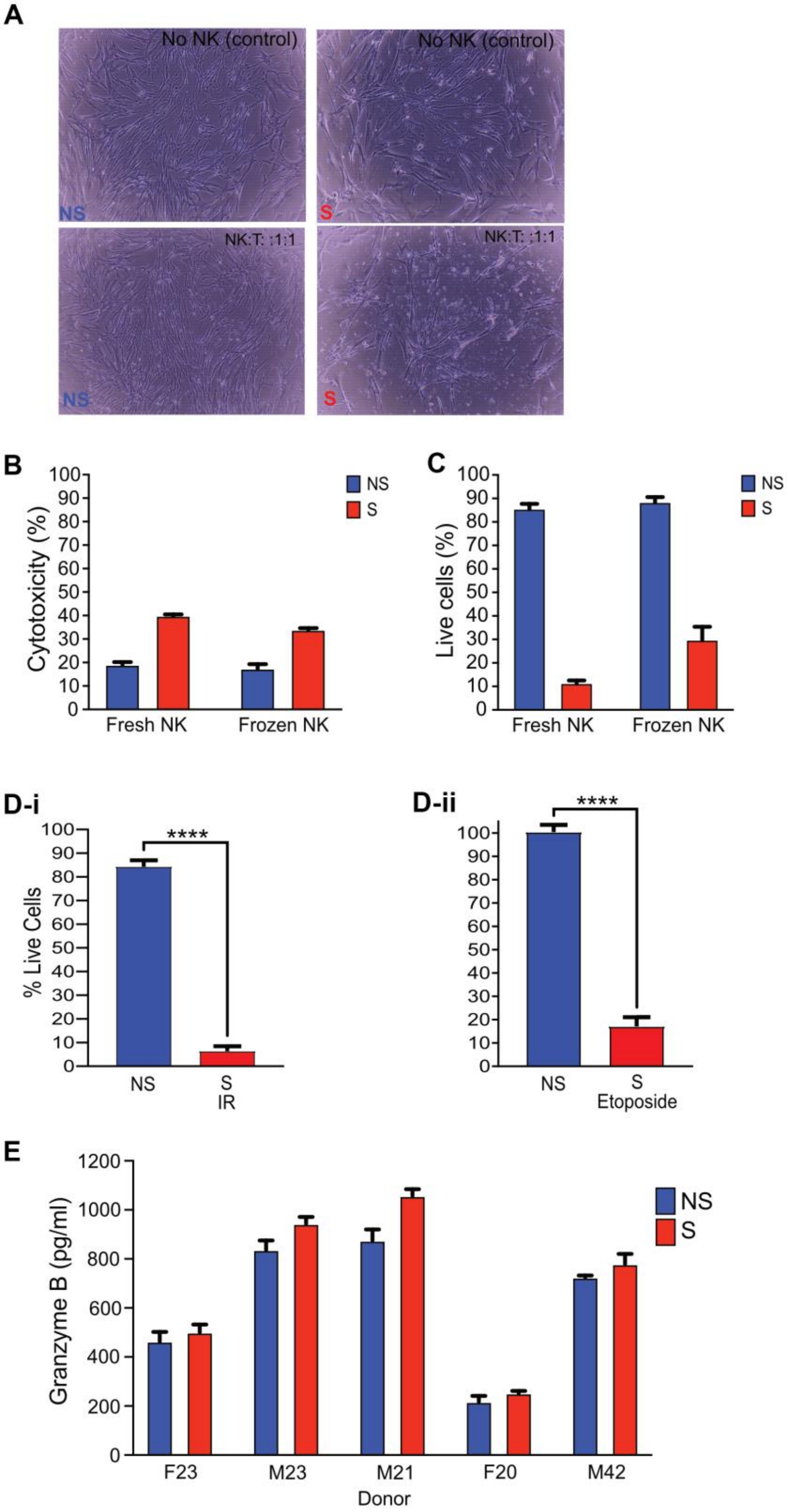


**Supplementary Figure 3. Activated primary NK cells selectively eliminate senescent cells.** Related to Figure 3. (**A**) Representative light microscopy images showing NK cell cytotoxicity towards NS versus S IMR-90 cells after 16 h of co-culture. (**B**) Cytotoxicity of NK cells that had been freshly isolated 3 d before co-culture("fresh")versusNKcellsthathadbeenrevived from storage in liquid nitrogen 3 d before co-culture ("frozen")after16hofco-culture. Cytotoxicity was evaluated by LDH release. (**C**) Quantitative analysis of IMR-90 target cell viability after 4 d of co-culture with frozen or fresh NK cells, as measured by Calcein AM. (**D-i**) Percentage of live cells after 4 d of co-culture with NS cells or fibroblasts induced to senesce by irradiation (20 Gy) or (**D-ii**) etoposide (20 μM, 48 h). (**E**) Granzyme B production of NK cells from 5 different donors in response to 16 h of incubation with NS or S IMR-90 cells. Donor sex and age are indicated in the figure.
